# Metabolomic Characterisation of Low-Density Lipoproteins Isolated from Iodixanol and KBr-Based Density Gradient Ultracentrifugation

**DOI:** 10.3390/metabo15020068

**Published:** 2025-01-22

**Authors:** Richard J. Webb, John K. Lodge, Sophie S. Scott, Ian G. Davies

**Affiliations:** 1 School of Health and Sport Sciences, Liverpool Hope University, Liverpool L16 9JD, UK; 2 School of Human Sciences, London Metropolitan University, London N7 8DB, UK; 3 Department of Applied Sciences, Northumbria University, Newcastle-Upon-Tyne NE1 7ST, UK; 4 Research Institute of Sport and Exercise Sciences, Faculty of Science, Liverpool John Moores University, Liverpool L3 3AF, UK

**Keywords:** lipoprotein, KBr, iodixanol, LC-MS, LDL, ultracentrifugation, metabolomics

## Abstract

Background/Objectives: Salt-based density gradient ultracentrifugation (SBUC) is frequently used to isolate lipoproteins for their subsequent analysis. However, the addition of salts may disrupt their molecular composition. Therefore, the aim of the present study was to assess the impact of SBUC upon the molecular composition of low-density lipoprotein (LDL) particles, compared to a validated non-salt method involving iodixanol gradient ultracentrifugation (IGUC). Methods: Whole human plasma was analysed for various lipid parameters before LDL particles were isolated using both SBUC and IGUC methods. Each fraction was then filtered to obtain low-molecular-weight compounds. The LDL molecular content of the resulting fractions from both methods was determined using untargeted liquid chromatography–mass spectrometry (LC-MS) in positive and negative modes. Results: A total of 1041 and 401 features were putatively identified using positive and negative modes, respectively. Differences were shown in the molecular composition of LDL prepared using SBUC and IGUC; in positive mode ionisation, the PLS-DA model showed reasonable fit and discriminatory power (R2 = 0.63, Q2 = 0.58, accuracy 0.88) and permutation testing was significant (*p* < 0.001). Conclusions: The findings reveal distinct differences in the small molecule composition of LDL prepared using the two methods, with IGUC exhibiting greater variation. In negative mode, both methods detected phospholipids, long-chain sphingolipids, and ceramides, but IGUC showed higher fold differences for some phospholipids. However, in positive mode, non-native brominated adducts were found in LDL isolated using SBUC and evidence of potential bacterial contamination was discovered in samples prepared using IGUC, both of which have the capacity to affect in vitro experiments.

## 1. Introduction

Lipoproteins are a diverse species of particles and are a rich source of polar and non-polar molecules [1,2,3]. Advances in metabolomic techniques allow for the analysis of a plethora of molecules with biological processes above and beyond traditional lipid metabolism [1,4]. Small non-polar and amphipathic molecules found within the interior and phospholipid surface monolayer of lipoprotein particles have garnered attention in recent years due to their wide-ranging functions. Using salt-based gradient ultracentrifugation (SBUC), various phospholipids and sphingolipids across different lipoproteins in different populations have been detected [5,6,7], showing involvement in various metabolic pathways, highlighting their potential diversity and metabolic importance. For example, higher levels of sphingolipids and ceramides with lower levels of phosphatidylcholines, located on the surface of LDL, increased the susceptibility of LDL aggregation [8]. This is a key step in the pathogenesis of atherosclerotic cardiovascular disease, which is modifiable with dietary intervention, both negatively with, e.g., saturated fat [8], and positively with *n-3* fatty acids [9]. Furthermore, differences in the severity of carotid lesions have recently been shown to be dependent on the LDL lipidome [10]. Furthermore, HDL isolated from patients with chronic kidney disease containing saturated and monounsaturated phosphatidylcholines, ceramides, and sphingomyelins with long-chain fatty acids was associated with increased all-cause mortality [11]. Similarly, HDL particles separated using SBUC from patients who had experienced an acute ST-segment elevation myocardial infarction were associated with increased proinflammatory lysolipids and alterations to intermediate-to-long-chain unsaturated phospholipid and sphingolipid species [12]. Conversely, a decrease in proinflammatory proteins was found in HDL isolated using SBUG from a single patient with severe COVID-19 who was administered with recombinant HDL, highlighting the potential utility of these insights to inform clinical practice [13].

The majority of lipidomic studies concerned with lipoproteins have focused on small non-polar molecules associated with the interior or the phospholipid surface monolayer [1,5]. To the authors’ knowledge, only one study has investigated additional small polar molecules (SPMs) attracted to, and non-covalently bonded to, the negative electrostatic charge on the lipoprotein surface [2]. This study analysed the VLDL, IDL, and LDL classes and HDL subclasses separated using SBUC showing several diverse SPMs, such as fatty acids, lactic acid, glucose, pentitol, and gulonic acid, attached to the surface of the HDL subfractions. Many of these were shown to correlate with insulin resistance, waist circumference, and glycolytic pathways, highlighting the far-reaching influence of these lipoprotein-associated molecules.

SBUC remains the most used lipoprotein separation method, likely due to its ubiquity in laboratories and well-defined, easily implemented SBUC protocols. SBUC requires the addition of either NaBr or KBr [14,15,16,17], offering a preparative separation for a wide range of analyses (e.g., traditional lipids, apolipoproteins, and ‘omics’). However, its ionic nature generates a hyperosmotic environment surrounding the lipoproteins, which induces a loss of water, increases their density, and disrupts the associated apolipoproteins [18], potentially leading to the dissolution of small polar molecules (SPMs). In addition, the increased centrifugation times (for some methods) can lead to a loss of associated proteins and alterations of the lipid components rendering LDL more susceptible to oxidation, and increased exposure to shear forces can dissociate apolipoproteins [19,20]. The use of D_2_O has been proposed as an alternative density gradient medium, with the composition of VLDL and LDL fractions being identical to those separated using KBr [21], and this method has been recently used with success to compare the molecular composition of LDL against the liver lipidome, revealing positive relationships with dihydroceramides and ceramides [22]. Furthermore, Ståhlman et al. [23] compared ionic KBr and a non-ionic combination of sucrose/D_2_O and found that sucrose/D_2_O yielded higher total protein and apolipoprotein levels in both HDL and LDL. Despite this, sucrose’s hygroscopic nature could affect lipoprotein hydration, making it less ideal for SPM analysis, and D_2_O may be prohibitively costly for some applications. We previously developed a method of separating LDL using a non-ionic density gradient medium, iodixanol gradient ultracentrifugation (IGUC) [24]. Iodixanol is inert, non-toxic, non-osmotic, and non-hygroscopic [25], and it separates LDL with a lower density [24,26], which is suggestive of maintaining a native hydration status. Furthermore, IGUC times are often shorter than those outlined in SBUC methods, which reduces exposure to shear forces.

Currently, there is a dearth of research investigating small molecules in the light of different density gradient media for lipoprotein ultracentrifugation. We aimed to address this by comparing a commonly used method of lipoprotein separation using potassium bromide (KBr) ultracentrifugation [17] with our previously validated method using iodixanol [24], which we hypothesised to be less disruptive to the small molecule composition of low-density lipoprotein (LDL). We hypothesise a unique ‘lipoprotein-omic’ approach using a superior non-ionic ultracentrifugation method combined with liquid chromatography–mass spectrometry (LC-MS) to investigate the presence and identity of these molecules in lipoproteins with a greater degree of sensitivity and, therefore, increase the potential for biomarker discovery.

## 2. Materials and Methods

### 2.1. Reagents

Optiprep (iodixanol), KBr, and Amicon ultra-centrifugal filters with a 30 Da cutoff were purchased from Sigma (St. Louis, MO, USA). For the analysis of plasma and lipoprotein classes, total cholesterol, triglycerides, HDL-C, small dense LDL (sdLDL), and apolipoprotein B (apoB) kits were purchased from Randox Laboratories Ltd. (Crumlin, UK). OptisealTM (11.2 mL) ultracentrifuge tubes were purchased from Beckman-Coulter (Brea, CA, USA).

### 2.2. Participant Recruitment

Healthy male volunteers (*n* = 21) were recruited from Liverpool John Moores University (LJMU) via emails and posters, as well as verbally.

### 2.3. Inclusion/Exclusion Criteria

All potential participants completed a screening questionnaire prior to consenting. Any potential participants who were using lipid-lowering medication or who had taken part in any other study over the previous 3 months which may have influenced their lipid profile were excluded from the study. Smokers were also excluded, as were participants using electronic implants, e.g., cardiac pacemakers, active prostheses, electronic life-support systems, e.g., artificial hearts, artificial lungs, or portable electronic medical devices, e.g., ECGs.

### 2.4. Ethical Approval

Ethical approval was granted by LJMU research ethics committee (REC Number: 16/ELS/012) following the principles of the Declaration of Helsinki for the use of human participants. Informed consent was obtained from all subjects involved in the study.

### 2.5. Participant Characteristics

Participants were characterised by age, body mass, and BMI using standard anthropometric methods. Body composition (body fat percentage, fat-free mass, etc.) was analysed using a SECA MBca 515 bioimpedance scale (SECA, Hamburg, Germany). Plasma samples were analysed for total cholesterol, triglycerides, apoB, sdLDL, and HDL-C using a Randox Daytona autoanalyser (Randox, Crumlin, UK).

### 2.6. Collection and Preparation of Plasma and Ultracentrifugation Methods

A blood sample was drawn from each participants’ antecubital vein using an EDTA-coated vacutainer tube. Plasma was then separated from the blood using low-speed centrifugation as described previously [24]. Plasma was separated into LDL and its subclasses using iodixanol and potassium bromide (KBr) lipoprotein separation methods. The methods of Davies et al. [24] and Chung et al. [17] were employed to separate LDL using IGUC and an adapted KBr SBUC, respectively. Both methods involved centrifugation for 3 h at 341,000 g_(av)_ in a Beckman Coulter Optima XPN-80 ultracentrifuge and a Beckman Coulter NVT-65 rotor (Beckman Coulter, Brea, CA, USA) with acceleration programme 5 and deceleration programme 5.

### 2.7. Fractionation and Determination of LDL

The resulting samples from both methods were fractionated using a Labconco Auto 147 Densi-Flow 115V (Labconco, Kansas City, MO, USA) and a Gilson FC203B fraction collector (Gilson Inc., Middleton, WI, USA) to generate 20 fractions of ~500 µL [17]. A proportion (circa 20 µL) of each fraction was analysed for its refractive index using an Abbe refractometer (Bellingham and Stanley, Basingstoke, UK). The refractive indices were converted to density using the following formula: ρ = ηa – b. Here, a = 3.2984, b = 3.3967, η = refractive index, and ρ = density [24]. For standard characterisation a small portion (circa 180 µL) of whole plasma and gradient fractions were analysed for cholesterol using a Randox Daytona autoanalyser (Randox, Crumlin, UK). LDL was determined using previously described cutoffs [17,24].

### 2.8. Preparation of Isolated LDL Samples

LDL samples were filtered through molecular weight centrifugal filters (30 Da) as per the manufacturer’s instructions. Each sample was centrifuged for 30 min at 10 degrees, and the resulting filtrate was then used for metabolomic analysis. Briefly, 50 µL of the filtrate was taken and mixed with an equivolume of chilled methanol (LC-MS pure), mixed and centrifuged (10,000 rpm) to remove any pellet, and then the supernatant was transferred to a sample vial for analysis.

### 2.9. Metabolomic Analysis of LDL Samples

Hydrophilic interaction liquid chromatography (HILIC)-based analysis was conducted using ultra high-resolution liquid chromatography (UHPLC) and mass spectrometry (MS) [27]. Metabolite profiles were generated on a Dionex 3000 ultra high-pressure liquid chromatography (UHPLC) system hyphenated to a Q-Exactive classic high-resolution mass spectrometer system (ThermoScientific, Bremen, Germany). All solvents and ionisation agents used were of analytical grade or higher unless stated. The chromatographic separation was performed on a Water Acuity Ethylene Bridge Hybrid Amide analytical column (2.1 × 150 mm) with a particle size of 1.7 micron at a flow rate of 400 µL/min; the column temperature was set to 45 °C. The binary buffer system was as follows: Buffer A was MilliQ water and Buffer B was acetonitrile, both with 10 mM ammonium formate adjusted to pH 3.5 using formic acid.

The LC profile was as follows: T:0 min: 90% (B); T:2 min 60% (B); T:5 min 40% (B); T:7.5 min 40% (B); T:7.6 min 90% (B); T:10 min 90% (B). A 3 µL injection was applied. The heated spray ionisation source (HESI) was set to the following parameters: a sheath gas flow rate of 50, an aux gas flow rate of 13, and a sweep gas flow rate of 3. The spray voltage was set to 3.5 akV with a Capillary temperature of 275 °C. The aux gas heater temperature was adjusted to 425 °C. The mass (MS1) acquisition range was as follows: 75–1000 *m*/*z* units at a mass resolution of 35,000 at approximately 7.6 scans per second, a microscan of 1, and the lock mass off. The AGC was set to 1 × 10^6^ and the ion injection time was 100 mS^−1^. The data were acquired on both positive and negative mode polarity (independently); the setting for the negative mode was the same as that for positive ion mode except the voltage was set to 2.5 kV. The system was primed with a minimum of 10 sequential injections of pooled QC to stabilise the HESI and to check for chromatographic stability before initialling the batch analysis. All samples were analysed with relevant QCs and in a randomised order to reduce any time effects of the analysis.

Peak table generation and alignment were performed using Compound Discoverer 2.1 (ThermoScientific, Bremen, Germany) with an alignment window of 0.25 min, a mass tolerance of 5 ppm, and a signal intensity threshold of 200,000 counts with a signal-to-noise ratio of 5:1.

### 2.10. Characterisation and Identification of Discriminating Features

Peak intensity tables from Compound Discoverer were processed using MetaboAnalyst© software, version 3 [28]. The full dataset was autoscaled. MetaboAnalyst performed detailed multivariate and univariate analyses, including Principal Component Analysis, which was used for data quality control, and Partial Least Squares Discriminant Analysis (PLS-DA), which was used to test for discrimination between sample groups. Cross-validation tests were used to test the robustness of the model, using Q2, R2, and classification metrics, while Variable Importance in Projection (VIP) data were used to rank the most discriminatory species. Annotation of the identified metabolites was carried out according to level 2 of the identification proposed by the Metabolomics Standards Initiative [29]. Firstly, the top discriminating features (VIP > 1.7, fold change > 0.19) in each mode underwent putative identification of these features, which was performed using the established database UCSD Metabolomics Workbench [30] and the Human metabolite database [31].

## 3. Results

### 3.1. Participant Characteristics

A total of 21 otherwise healthy males (mean age 39.0 years) participated in the study and had a mean BMI of 26.5 kg/m^2^ and a waist circumference of 93.0 cm, as described in Table 1. Participants also had a mean average of 2.4 L of visceral adipose tissue. In terms of the standard lipid profile, all markers were in the normal range apart from total cholesterol, which was slightly elevated at 5.32 mmol/L. Apolipoprotein B was 72.81 mg/dL and sdLDL was 0.75 mmol/L. Finally, plasma glucose levels were 5.82 mmol/L and mean plasma protein was 76.03 g/L.

### 3.2. Metabolite Profiling

**Figure 1:**
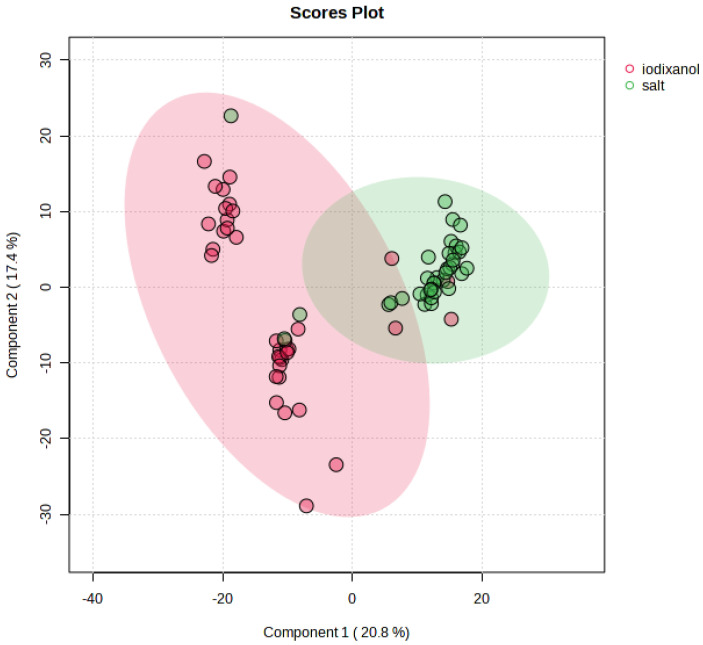
PLS-DA score plot comparing the LDL fractionation methods, in positive mode ionisation.

**Figure 2:**
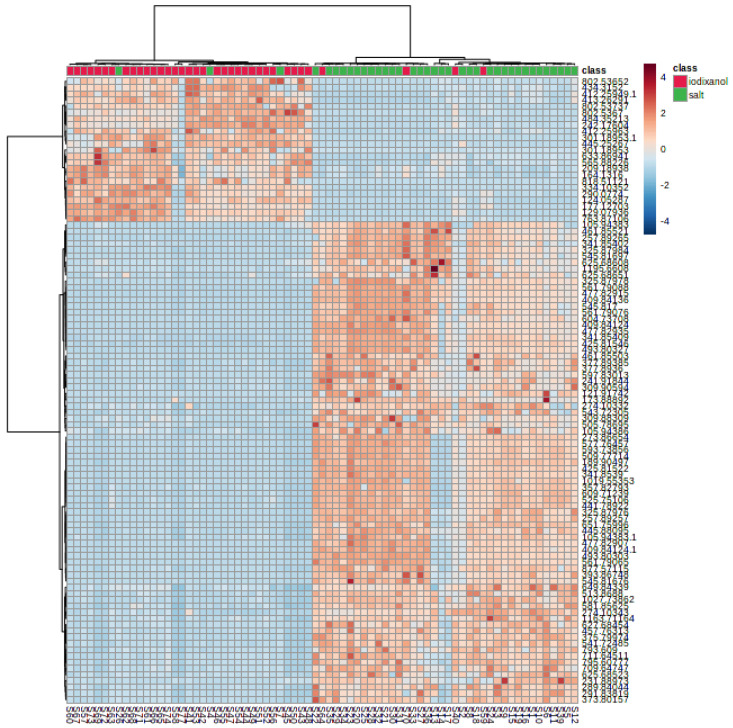
Heat map of top discriminatory features for each treatment class, in positive mode.

Samples containing LDL from both IGUC and SBUC fractionation were compared. The analysis identified 1041 and 401 features in positive and negative mode, respectively. Initially, PCA was used to identify any outlying samples, but none were found, so we proceeded to establish supervised models of discrimination between the fractionation methods. Figure 1 shows a PLS-DA score plot of a representative analysis of LDL comparing the fractionation methods, in positive mode ionisation. The model shows reasonable fit and discriminatory power (R2 = 0.63, Q2 = 0.58, accuracy 0.88). Permutation testing (1000 permutations) on prediction accuracy was significant (*p* < 0.001). Although samples from IGUC and SBUC fractions were clustered separately, those from iodixanol display a larger variation through component 2 and were separated into distinct clusters not seen in the SBUC fractions. All samples were within Hotelling’s confidence band, even though certain samples appeared in the other cluster. A heat map of the top 100 discriminatory features (Figure 2) shows similar intensity profiles for each treatment class. The heat map also highlights that certain sample profiles are more representative of the opposite treatment. Similar results were found in negative mode.

Table 2 and Table 3 show the top discriminatory species in each ionisation mode with a VIP either >2.0 (positive mode) or >1.7 (negative mode). To putatively identify discriminatory ions, closest matches were obtained from metabolite databases, and these preliminary identities are also shown in Table 2 and Table 3. Therefore, metabolite identifications are level 2 of the Metabolomics Standards Initiative [29]. There is limited crossover of preliminary annotations between ionisation modes with no single species predominating. However, negative mode did highlight lipid species, such as analogues of PA and PE, whilst positive mode highlighted certain bromine adducts.

## 4. Discussion

The primary aim of this study was to develop and apply a novel ‘lipoprotein-omic’ approach to investigate lipoprotein metabolite composition, comparing IGUC and SBUC by employing LC-MS. We hypothesised that IGUC would maintain native hydration status in LDL separation while challenging the commonly used SBUC LDL density method. We show here that LDL isolated from the two treatments have very different metabolite profiles.

We found that LDL samples fractionated using IGUC exhibited greater variation along component 2 and were separated into distinct clusters. This clustering pattern was not observed in the samples fractionated by salt, indicating a difference in the two methods, although it should be noted that the origin of the molecules is unknown. The intensity profiles of the samples were generally consistent within each treatment class (Figure 2), but certain samples displayed characteristics more representative of the opposite treatment, highlighting some overlap between the fractionation methods.

This overlap was shown when using negative ion mode, as both UC methods identified a range of phospholipids on the surface of LDL particles, e.g., PE and PA, that contribute to the structural integrity, fluidity, lipid transfer, and molecular interactions of LDL [32]. We also found lipid species consistent with long-chain sphingolipids or ceramides, known for enhancing LDL stability, supporting membrane structure, and participating in cellular signalling, with potential roles in modulating inflammatory and aggregation responses [8]. Other studies have also shown the presence of these phospholipids in LDL particles, emphasising their importance in identifying cardiovascular disease risk above and beyond LDL-C [3,33]. Furthermore, it has also been shown that some molecules found on LDL, such as sphingolipids, are positively associated with corresponding species found in the liver lipidome, suggesting that molecular inter-relations may extend beyond LDL particles themselves [22].

We also putatively identified key discriminatory ions based on VIP and fold changes in ion intensities, which differed between treatment groups. These included phosphatidylethanolamines, the amino sugar UDP-2-acetamido-2,6-dideoxy-beta-L-talose, and peptides which were detected in the IGUC samples when using negative ion mode, and they were all found to have a fold change >4. The higher fold difference in some phospholipids (e.g., PE/Cerp) observed with IGUC suggests that SBUC may facilitate the dissociation of these lipids from LDL particles, potentially due to the disruptive effects of high salt concentrations on lipid–lipoprotein interactions. Similarly, in positive ion mode, N-(2-Pyrimidinyl)formamide was also found to be higher in the IGUC treatment group. Despite having rather low fold change values, several brominated adducts were also identified in the SBUC treatment group only. It is suspected that these compounds may have been formed during the separation process and result from interactions between KBr, which was added as a density gradient medium in supraphysiological concentrations, and molecules within plasma. These adducts are not native to LDL but need to be considered when preparing lipoproteins for subsequent in vitro experiments as they may potentially distort results. To the authors’ knowledge there are no studies that have specifically investigated the impact of these molecules in LDL in vitro experiments. However, as studies investigating LDL typically address myriad research questions, such as cellular uptake, oxidation, glycation, aggregation, other modifications, and DNA alterations [8,33,34], they provide ample opportunities for brominated compounds to potentially interfere. This is supported by evidence from pharmaceutical research which has shown that brominated nucleotides have the ability to damage DNA [35]. There is little research to draw upon specifically regarding LDL, but we speculate that isolating these lipoproteins using SBUC may affect subsequent in vitro experiments, potentially distorting results. This is reflected in the evidence which does exist; for example, Canclini et al. compared an SBUC against IGUC, the precipitation of apoB containing lipoproteins and fast protein liquid chromatography, for separation of LDL and the subsequent analysis of proprotein convertase subtilisin/kexin type-9 (PCSK9) [36]. The findings revealed heterogeneity between methods, with PCSK9 appearing to be sensitive to SBUC but not IGUC, which may have direct consequences for future investigations aiming to further elucidate the function of PSCK9 as well broader potential implications for any experiments requiring the isolation of LDL using ultracentrifugation [36].

In contrast, the IGUC method, unsurprisingly, did not result in the formation of brominated adducts, preserving LDL in its native-like state. This is important, as phospholipids such as phosphatidylethanolamine and phosphatidic acid, along with sphingolipids and ceramides, are increasingly being recognised as biomarkers for cardiometabolic and cardiovascular disease [37,38]. Most studies, however, have focused on whole plasma/serum, with only a small number of studies utilising LDL. For example, a recent study investigated the impact of phosphor- and sphingolipids and, in the LDL particle, showed higher levels of certain phosphatidylethanolamines and sphingomyelins with more severe cases of carotid lesions [10]. Additionally, understanding the downstream effects of metabolic disease is vital for gaining further insights regarding atherosclerotic cardiovascular disease (ASCVD). In this respect, Lahelma et al. [22] showed that in patients with obesity that the liver and LDL lipidomes are correlated, reflecting hepatic lipid metabolism and its influence on LDL composition. This suggests that alterations in hepatic lipid metabolism due to obesity may directly contribute to the atherogenic potential of LDL particles, providing a mechanistic link between metabolic dysfunction and ASCVD progression. These studies highlight the importance of preserving the LDL lipidome as close to its native-like state, which therefore enhances the utility of the IGUC method in cardiometabolic research. In contrast, SBUC’s potential disruption of these lipids could lead to the loss of valid information about LDL’s role in disease pathways. Therefore, the choice of separation method may directly impact the reliability of the results.

However, IGUC does present its own concerns, with evidence of bacterial contamination. We found UDP-2-acetamido-2,6-dideoxy-beta-L-talose in negative mode with IGUC; this is a microbial lipopolysaccharide (LPS) by-product which may possibly suggest contamination during sample handling or potentially in vivo LPS translocation from the gut into the blood where binding to LDL may have occurred. The potential detection of LPS on LDL, whether in vitro or in vivo, has possible implications since LPS binding can modify LDL’s oxidative state and inflammatory properties [39,40], potentially influencing findings related to LDL’s function in cardiovascular health and immune interactions. SBUC may be a better choice to avoid contamination, as the high salt gradient inhibits bacterial growth and reduces the risk of microbial by-products, making it more suitable for studies where LPS binding or bacterial contamination could affect results, particularly in inflammation or immune-related LDL research. On the other hand, this finding could also represent an opportunity to explore novel LDL–LPS interactions, particularly in the context of gut-derived inflammation and its connection to cardiovascular risk. In endotoxemia, LPS binds to lipoproteins, including LDL in the blood, and this interaction is influenced by its phospholipid content [41]. IGUC may offer a more accurate platform for studying these interactions by preserving the native PL composition of LDL, enabling better insights into lipoprotein–LPS interactions.

Further research is required to confirm the presence of novel metabolites located on LDL, including the source of bacterial contamination. To further improve the accuracy of experiments using IGUC, implementing stricter sterile handling protocols, filtering plasma samples before ultracentrifugation, using antimicrobial treatments, and performing quality control checks for contamination before and after separation to minimise bacterial presence are recommended.

Other common consistencies found in the IGUC samples were that identified compounds generally had higher fold changes and greater molecular weights, particularly in negative mode. That said, whilst we appreciate that the metabolite assignments are putative (level 2 of the Metabolomics Standards Initiative), there did not appear to be any additional patterns in the class of metabolites predominating in either treatment. Furthermore, the overlap in the discriminatory ions between positive and negative ionisation modes was limited, with no single species dominating across both.

## 5. Limitations

There are several limitations with our study that should be acknowledged. The sample size was small and homogeneous, with only 21 healthy male participants. While we provide valuable insights, the lack of diversity limits the generalisability of the findings to broader populations, including females and individuals with different health conditions or lipid profiles. Future studies should include larger and more representative cohorts to validate these results and assess their relevance across a wider range of populations, including those with cardiovascular diseases or other metabolic disorders.

Both ultracentrifugation methods used in the study present inherent limitations. While IGUC appears to preserve LDL in a more native-like state, the risk of bacterial contamination remains a concern. On the other hand, SBUC avoids contamination but results in the formation of non-native brominated compounds that may be disruptive regarding in vitro experimental work. These methodological trade-offs suggest that neither method is without shortcomings, and further optimisation of LDL separation techniques is needed to ensure more accurate and reliable results. It should also be noted that environmental attributes, including temperature and pressure, have previously been shown to affect the visibility of plasma lipids, lipoproteins, and their subclasses after isolation via ultracentrifugation and analysis using nuclear magnetic resonance spectroscopy [42]. Attributes such as these were not robustly accounted for in the present study and should be considered for future investigations.

The study also relied on the putative identification of metabolites based on established databases and followed level 2 of the Metabolomics Standards Initiative. While these findings provide valuable initial insights, they remain tentative. Confirmatory studies using more targeted approaches, such as tandem mass spectrometry (MS/MS) or isotopically labelled standards, are required to validate the identity of the metabolites and confirm their physiological relevance.

Additionally, the robustness of the study’s findings could be strengthened through more extensive cross-validation. While cross-validation tests were used to evaluate the PLS-DA model, further validation using larger and more diverse datasets would enhance confidence in the results and improve their applicability to broader populations.

## 6. Conclusions

Both UC methods putatively identified a range of phospholipids and long-chain sphingolipids/ceramides on the surface of LDL, highlighting the utility of these methods for investigating LDL’s role in cardiovascular disease risk beyond LDL-C. However, both methods have limitations, with SBUC uniquely leading to the formation of brominated adducts, which are not native to LDL. In contrast, IGUC preserved LDL in a more native-like state with higher fold differences for some phospholipids but presented challenges with potential bacterial contamination. Both methods could impact in vitro experiments by altering LDL’s interactions. While further work is needed to confirm our findings, we recommend using sterile protocols and quality control to ensure the accuracy and reliability of LDL preparation.

## Figures and Tables

**Table 1:** Participant characteristics and metabolic markers.

Variable	Mean ± SD
Sex	All male
Age	39.0 ± 12.2
Height (m)	1.77 ± 0.1
Weight (kg)	82.5 ± 8.9
BMI (kg/m^2^)	26.5 ± 3.0
Fat mass (kg)	20.3 ± 7.3
Fat mass (%)	24.2 ± 7.1
Fat-free mass (kg)	62.2 ± 5.7
Fat-free mass (%)	75.7 ± 7.2
Visceral adipose tissue (L)	2.4 ± 1.1
Waist circumference (cm)	93.0 ± 10.0
Triglycerides (mmol/L)	1.07 ± 0.47
Total cholesterol (mmol/L)	5.32 ± 1.20
LDL-C (mmol/L)	3.00 ± 1.05
HDL-C (mmol/L)	1.36 ± 0.33
Apolipoprotein B (mg/dL)	72.81 ± 22.27
sdLDL (mmol/L)	0.75 ± 0.38
Glucose (mmol/L)	5.82 ± 0.61
Total protein (g/L)	76.03 ± 3.12

Abbreviations: BMI, body mass index; LDL-C, low-density lipoprotein cholesterol; HDL-C, high-density lipoprotein cholesterol; sdLDL, small dense LDL.

**Table 2:** Top discriminatory species, positive mode.

*m*/*z*	VIP (>2)	t.stat	*p*-Value	FDR	Highest In	Fold Change	Top Match (M + H, 15 ppm Tolerance)	Formula
425.81546	2.1308	−9.8971	5.28 × 10^−15^	4.38 × 10^−12^	salt	0.19	8-bromo-adenosine-5”monophosphate	C_10_H_14_N_5_O_7_PBr
124.05287	2.1207	9.7866	8.41 × 10^−15^	4.38 × 10^−12^	iodixanol	4.21	N-(2-Pyrimidinyl)formamide	C_5_H_5_N_3_O
561.79088	2.0985	−9.5502	2.28 × 10^−14^	6.70 × 10^−12^	salt	0.19	No match	
1027.7386	2.0957	−9.5213	2.57 × 10^−14^	6.70 × 10^−12^	salt	0.28	PIP(PGF1alpha/18:0)	C_47_H_88_O_19_P_2_
581.85625	2.0815	−9.3765	4.75 × 10^−14^	9.89 × 10^−12^	salt	0.34	Nakamuric acid	C_20_H_22_N_7_O_4_
513.8688	2.0695	−9.2563	7.90 × 10^−14^	1.24 × 10^−11^	salt	0.27	2,5-Diaminopyrimidine nucleoside triphosphate	C_9_H_19_N_5_O_14_P_3_
341.85409	2.0621	−9.1846	1.07 × 10^−13^	1.24 × 10^−11^	salt	0.19	Cyano-6,8-dibromo-4-methylcoumarin	C_11_H_6_NO_2_
457.76313	2.0441	−9.0112	2.24 × 10^−13^	2.33 × 10^−11^	salt	0.25	Convolutamine A	C_13_H_19_NO_2_
445.88095	2.0397	−8.9701	2.67 × 10^−13^	2.52 × 10^−11^	salt	0.26	6-Bromo-3,4-di(4′-chlorophenyl)coumarin	C_21_H_12_O_22_
377.89385	2.0235	−8.8204	5.04 × 10^−13^	4.04 × 10^−11^	salt	0.24	(3S)-3-(2,3-Dibromo-4,5-dihydroxybenzyl)pyrroline	C_11_H_10_NO_4_
525.75106	2.0147	−8.7399	7.11 × 10^−13^	4.35 × 10^−11^	salt	0.20	3,3′-Diiodothyronine	C_12_H_14_NO_5_
289.84044	2.0109	−8.7058	8.22 × 10^−13^	4.75 × 10^−11^	salt	0.27	S-(4-Bromo-benzyl)cysteine	C_15_H_14_NO_4_
651.75996	2.0095	−8.6932	8.67 × 10^−13^	4.75 × 10^−11^	salt	0.23	2S,3R-didecanoyl-docosane-2,3-diol	C_10_H_13_O_2_S
325.87984	2.0062	−8.6646	9.80 × 10^−13^	4.86 × 10^−11^	salt	0.22	(4S)-2-Bromo-4-[(5R)-2-amino-4-oxo-2-imidazolin… TG derivative	C_20_H_17_O_5_S
375.79974	2.0013	−8.6204	1.18 × 10^−12^	5.60 × 10^−11^	salt	0.28	caelestine D	C_11_H_13_N_5_O_2_
593.73856	2	−8.6078	1.25 × 10^−12^	5.65 × 10^−11^	salt	0.19	Arachidyl arachidate	C_11_H_8_NO_4_

**Table 3:** Top discriminatory species, negative mode.

*m*/*z*	VIP (>1.75)	t.stat	*p*-Value	FDR	Highest In	Fold Change	Top Match (M-H, 15 ppm Tolerance)	Formula
517.82311	1.8786	−12.559	8.14 × 10^−20^	1.82 × 10^−17^	salt	0.28	14-Oxoaerophobin2	C_16_H_18_N_5_O_5_
601.78459	1.8768	−12.522	9.44 × 10^−20^	1.82 × 10^−17^	salt	0.29	Guanosine 3′,5′-bis(diphosphate)	C_10_H_16_N_5_O_17_P_4_
501.84957	1.869	−12.359	1.81 × 10^−19^	1.82 × 10^−17^	salt	0.26	Deltamethrin	C_22_H_18_NO_3_
585.8107	1.8689	−12.357	1.82 × 10^−19^	1.82 × 10^−17^	salt	0.27	Adenosine tetraphosphate	C_10_H_16_N_5_O_16_P_4_
533.79708	1.8586	−12.147	4.20 × 10^−19^	2.81 × 10^−17^	salt	0.33	Trimethylpentatriacontane	C_38_H_77_
617.75814	1.8553	−12.082	5.47 × 10^−19^	3.13 × 10^−17^	salt	0.32	methyl tricosanyl oleate	C_42_H_81_O_2_
569.83678	1.8441	−11.865	1.31 × 10^−18^	6.56 × 10^−17^	salt	0.23	Iopanoic acidD	C_11_H_11_NO_2_
433.86207	1.8341	−11.678	2.79 × 10^−18^	1.24 × 10^−16^	salt	0.29	Oxyclozanide	C_19_H_16_NO_5_S_3_
349.90199	1.8083	−11.221	1.81 × 10^−17^	5.59 × 10^−16^	salt	0.29	alpha-D-glucuronate 1-phosphate(Br adduct)	C_10_H_10_NO_3_
365.87555	1.8048	−11.161	2.32 × 10^−17^	6.64 × 10^−16^	salt	0.35	-(1,3-Benzoxazol-2-YL)-2,6-di-bromophenol	C_13_H_6_NO_2_
551.76936	1.7917	−10.944	5.69 × 10^−17^	1.52 × 10^−15^	salt	0.27	keronopsin A2	C_18_H_13_NO_6_S
719.6917	1.7898	−10.913	6.45 × 10^−17^	1.62 × 10^−15^	salt	0.23	hydroxyphthioceranic acid (C48)	C_48_H_95_O_3_
657.88869	1.787	10.868	7.78 × 10^−17^	1.84 × 10^−15^	iodixanol	5.30	No match	
856.85394	1.7828	10.802	1.02 × 10^−16^	2.28 × 10^−15^	iodixanol	11.74	PE/CerP	C_51_H_103_NO_6_P
1723.7027	1.7752	10.682	1.69 × 10^−16^	3.56 × 10^−15^	iodixanol	7.52	Microspinosamide	C_75_H_108_N_18_O_22_S
1315.7779	1.7646	10.52	3.32 × 10^−16^	6.65 × 10^−15^	iodixanol	5.22	PA derivative	C_68_H_111_N_6_O_19_
801.65486	1.7613	−10.469	4.11 × 10^−16^	7.48 × 10^−15^	salt	0.24	PA/PE/PG derivative	C_46_H_90_O_8_P
589.90225	1.7514	10.323	7.58 × 10^−16^	1.22 × 10^−14^	iodixanol	4.67	UDP-2-acetamido-2,6-dideoxy-beta-L-talose	C_17_H_26_N_3_O_16_P_2_
1587.7285	1.7513	10.321	7.63 × 10^−16^	1.22 × 10^−14^	iodixanol	6.79	No match	

## Data Availability

The original contributions presented in this study are included in the article. Further inquiries can be directed to the corresponding author.
